# Relationship between genetic polymorphism of drug transporters and the efficacy of *Rosuvastatin*, *atorvastatin* and *simvastatin* in patients with hyperlipidemia

**DOI:** 10.1186/s12944-021-01586-7

**Published:** 2021-11-08

**Authors:** Andrey Sivkov, Natalya Chernus, Roman Gorenkov, Sergey Sivkov, Svetlana Sivkova, Tamara Savina

**Affiliations:** 1 N.V. Sklifosovsky Institute of Clinical Medicine, Department of Clinical Pharmacology and Propaedeutics of Internal Diseases, I.M. Sechenov First Moscow State Medical University (Sechenov University), Moscow, Russian Federation; 2 N.V. Sklifosovsky Institute of Clinical Medicine, Department of Polyclinical Therapy, I.M. Sechenov First Moscow State Medical University (Sechenov University), Moscow, Russian Federation; 3 Institute of Leadership and Health Management, I.M. Sechenov First Moscow State Medical University (Sechenov University), Moscow, Russian Federation; 4 Federal State Budget Scientific Institution, «The N. A. Semashko National Research Institute of Public Health», Moscow, Russian Federation; 5 N.V. Sklifosovsky Institute of Clinical Medicine, Department of Polyclinical Therapy, I.M. Sechenov First Moscow State Medical University (Sechenov University), Moscow, Russian Federation

**Keywords:** Cardiovascular diseases, Hyperlipidemia, Cholesterol, Low-density lipoprotein, Hydroxymethylglutaryl-CoA reductase inhibitors, Pharmacogenetics

## Abstract

**Background:**

To determine the effect of genetic polymorphism of drug transporters on the efficacy of treatment with *Rosuvastatin*, *Atorvastatin* and *Simvastatin* in patients with hyperlipidemia.

**Methods:**

The study consists of 180 patients, aged 40–75 years, with hyperlipidemia. All patients were divided into two equal groups: patients with different SLCO1B1 (521CC, 521CT and 521TT) and MDR1 (3435CC, 3435TC and 3435TT) genotypes. Each group was divided into rosuvastatin-treated, atorvastatin-treated and simvastatin-treated subgroups. The lipid-lowering effect of statins was assessed by tracing changes in total cholesterol (TC) and low-density lipoprotein cholesterol (LDL-C) levels.

**Results:**

The use of statins over a 4-month period led to substantial reductions in TC and LDL-C levels. The hypolipidemic effect of studied agents was seen in both groups. However, it was less pronounced in patients with 521CC genotype. No statistically significantly differences were found between carriers of 3435TT, 3435CT and 3435CC genotypes.

**Conclusions:**

The lipid-lowering efficacy of rosuvastatin was higher compared to other two statins. Patients with *SLCO1B1* 521CC genotype are more likely to encounter a decrease in the hypolipidemic effect of statins. Such a risk should be considered when treating this category of patients. MDR1 polymorphism had no significant effect on statin efficacy.

## Introduction

Dyslipidemia and atherosclerosis are known to play a vital role in the development of coronary heart disease and arterial hypertension [1]. The atherosclerotic process is associated with elevated levels of cholesterol and low-density lipoproteins (LDLs) [2].

The main group of drugs used to treat dyslipidemia are statins [3, 4]. At present, the most commonly used statins are simvastatin, rosuvastatin and atorvastatin [5, 6]. The indication for statin therapy as primary prevention of cardiovascular complications is having a high level of cholesterol in the blood [7].

Statins are mandatory in the secondary prevention of myocardial infarction, and a high-dose statin therapy is justified in the treatment of patients with an acute coronary syndrome [8–10]. However, such treatment often has unwanted side effects, such as constipation, less often diarrhea, rhabdomyolysis, liver dysfunction, pancreatitis, myopathy, dizziness, and more [11–13]. Despite the proven efficacy of statins, their effectiveness and toxicity can vary from patient to patient [14]. The efficacy and side effects of statins can correlate with the presence of genetic polymorphisms, which affect the pharmacokinetics and pharmacodynamics of lipid-lowering agents. However, little studies in pharmacogenetics have been devoted to the identification of genetic markers for statin safety. Among the observations, one is that SLCO1B1 c.521 T > C polymorphism is most associated with myopathy and rhabdomyolysis [15–18]. Hence, more studies are required about the effect of genetic polymorphisms of transporters involved in lipid transportation on statin efficacy.

Organic anion (OATP) transporters actively capture and transport statins into hepatocytes. All statins are substrates for organic anion-transporting polypeptides 1B1 (OATP1B1), the transport activity of which is associated with the SLCO1B1 gene. At the same time, OATP1B1 is the only transporter of simvastatin. Rosuvastatin is a substrate for OATP1B3 and OATP2B1, and atorvastatin is a substrate for OATP2B1 [19]. Medical literature provides contradictory data on the effect of genetic polymorphism of OATP transporters on the lipid response to statin treatment in patients of different ethnicities. While some studies hold that it can affect the pharmacodynamic effect of statins [20–22], other studies give no evidence to support such an effect [23].

Hepatobiliary and renal-urinary transport of statins and their metabolites occurs largely through the ATP-binding cassette (ABC) transport protein P-glycoprotein (ABCB1), the transport function of which is associated with the MDR1 gene [24]. P-glycoprotein is responsible for the excretion of statins and prevents penetration into the systemic circulation. It also limits reabsorption by the kidneys. C3435T polymorphism in the MDR1 gene causes the dysfunction of P-glycoprotein, leading to variability in the pharmacokinetic profile of statins and, consequently, affects their effectiveness [24, 25].

Given the above, there is a need to investigate statin efficacy with respect to С521Т and С3435Т polymorphism in the SLCO1B1 and MDR1 genes. This study aims to determine the effect of genetic polymorphism of drug transporters on the efficacy of treatment with *Rosuvastatin*, *Atorvastatin* and *Simvastatin* in patients with hyperlipidemia.

## Materials and methods

The study of statin efficacy and genetic polymorphism involved 180 patients (43 male and 137 female) with hyperlipidemia (HDH), aged 40 to 75 years. Patients with these disorders were excluded from the study: secondary HDH, heart failure (NYHA class III–IV), symptomatic hypertension, elevated hepatic enzymes (> 50% above), kidney diseases (including kidney failure), and statin intolerance.

**Fig. 1:**
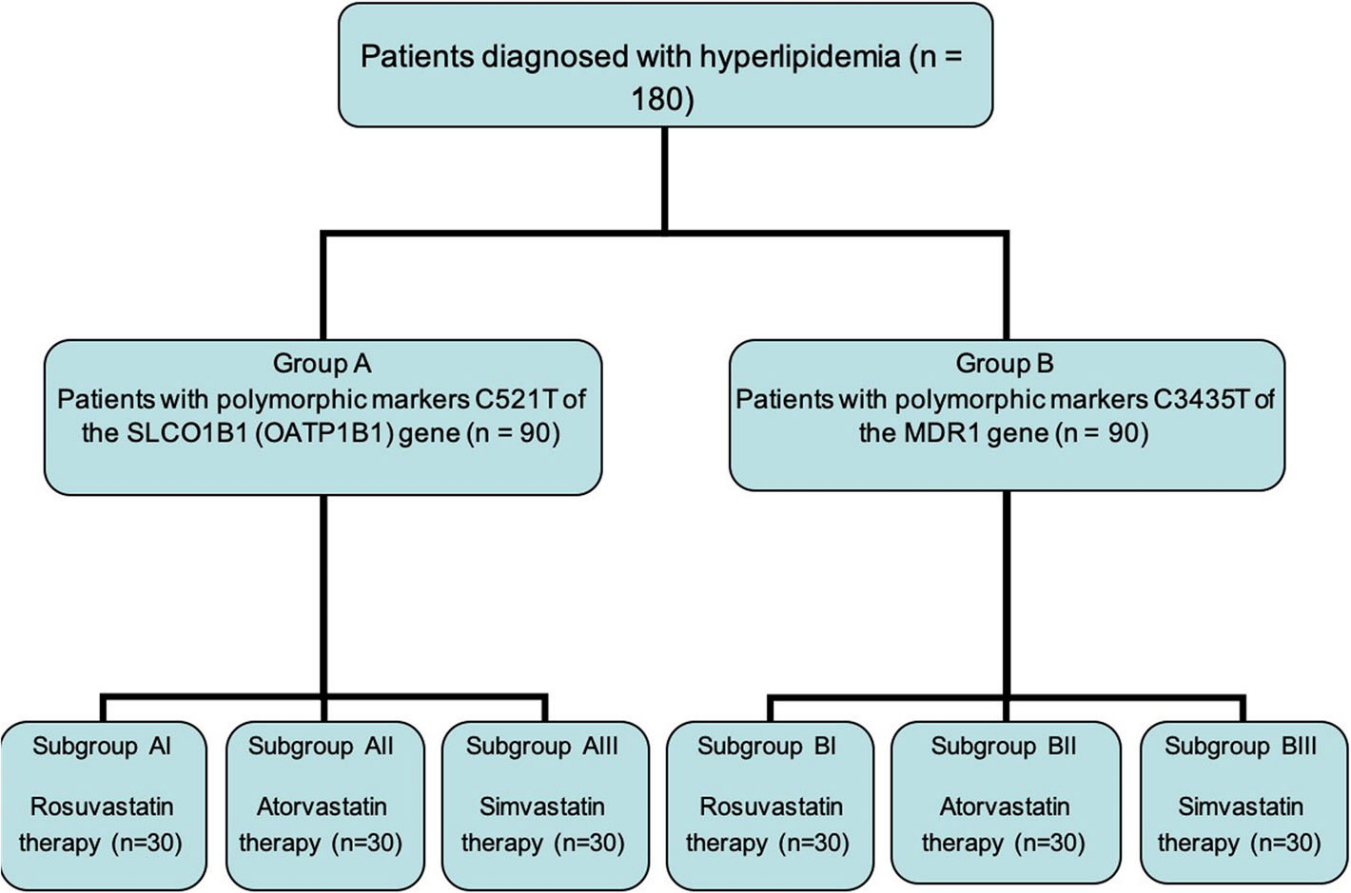
Flow chart showing the number of patients in each examined group who underwent rosuvastatin, atorvastatin and simvastatin treatment

The carriage of C3435T and T521C polymorphic variants was determined by a polymerase chain reaction-restriction fragment length polymorphism (PCR-RFLP) assay. Studies were performed on cubital vein blood samples. Based on the results of the assay, all patients were divided into two equal groups (Fig. 1).

Group A consists of patients with C521T polymorphism in the *SLCO1B1* gene, which encodes OATP1B1. Group B encompasses patients with *MDR1* C3435T genotypes. In each group, patients with different types of genotypes were randomly divided into three subgroups of 30 patients based on the type of drug they were given. Subgroups AI and BI received *rosuvastatin therapy*. Subgroups AII and BII received *atorvastatin therapy*. Subgroups AIII and BIII underwent *simvastatin therapy*. The details of clinical and demographic data of all patients are presented in Table 1.

**Table 1:** Demographic and clinical characteristics of patients with SLCO1B1 С521Т polymorphism (Group A) and MDR1 С3435Т polymorphism (Group B) who underwent statin treatment (Rosuvastatin, Atorvastatin and Simvastatin)

Characteristic	Rosuvastatin		Atorvastatin		Simvastatin	
	Subgroup A I (*n* = 30)	Subgroup B I (*n* = 30)	Subgroup A II (*n* = 30)	Subgroup B II (n = 30)	Subgroup A III (n = 30)	Subgroup B III (n = 30)
Sex, male/female	6 (20%)/ 24 (80%)	8 (26,6%)/ 22 (73,4%)	7 (23%)/ 23 (77%)	9 (30%)/ 21 (70%)	5 (16,7%)/ 25 (83,3%)	8 (26,6%)/ 22 (73,4%)
Average age, years (М ± m)	55.2 ± 1.47	54.7 ± 1.42	57.3 ± 1.59	58.2 ± 1.61	55.9 ± 1.63	56.8 ± 1.54
Hyperlipoproteinemia Type IIa	16 (53%)	18 (60%)	17 (56,7%)	19 (63,3%)	18 (60%)	20 (66,7%)
Hyperlipoproteinemia Type IIb	14 (46,7%)	12 (40%)	13 (43,3%)	11 (36,7%)	12 (40%)	10 (33,3%)
Arterial hypertension Degree I–II	25 (83,3%)	24 (80%)	29 (96,7%)	27 (90%)	30 (100%)	28 (93,3%)

The initial dosage of studied statins (Rosuvastatin, Atorvastatin and Simvastatin) was 10 mg per day. After four months of therapy, the dosage level was 40 mg per day.

The blood samples were used to measure the concentration of each of the following lipid parameters: total cholesterol (TC), triglycerides (TG), low-density lipoprotein cholesterol (LDL-C), very low-density lipoprotein cholesterol (VLDL-C), and high-density lipoprotein cholesterol (HDL-C). Lipid profiles were measured spectrophotometrically. HDL-C concentration was determined after precipitation of LDL and VLDL in blood serum using phosphotungstic acid. The lipid-lowering efficacy of statins with respect to genetic polymorphism of drug transporters was evaluated by the change in TC and LDL-C levels.

Statistical data analysis was carried out in EXEL using generally accepted methods. The paired Wilcoxon test was used to find statistically significant differences between baseline and post-treatment data. The Mann-Whitney test was used to measure the statistical significance of differences between baseline and follow-up levels of total and LDL cholesterol in subgroups. The statistical significance of differences between patients with different genotypes was established using one-way analysis of variance (ANOVA). Differences were considered statistically significant at *p* < 0.05. Absolute differences are presented as means with their standard deviations (M ± SD), and relative differences are expressed as percentages.

## Results

The distribution of the *SLCO1B1* C521T and *MDR1* C3435T polymorphism genotypes is shown in Table 2. Among the genotypes with the C521T polymorphic marker of the *SLCOB1* - OATP - C gene, there was a predominance of patients with the genotype 521TT. It makes up 50% of all cases in Subgroup AI, 53.3% of all cases in Subgroup AII, and 40% of all cases in Subgroup AIII. The 521CC and 521CT genotypes were less prevalent. Among the genotypes with the С3435Т polymorphic marker of the *MDR1* gene, there was a predominance of patients with the genotype 3435TC. It makes up 50%of all cases in Subgroup BI, 46.7% of all cases in Subgroup BII, and 50% of all cases in Subgroup BIII. The 521CC and 521CT genotypes were less prevalent.

**Table 2:** The prevalence of SLCO1B1 С521Т genotypes (Group A) and MDR1 С3435Т genotypes (Group B) in patients with HDH who underwent statin treatment (Rosuvastatin, Atorvastatin and Simvastatin)

Subgroup	Genotypes		
SLCO1B1 С521Т (Group A)			
	521СС	521ТТ	521ТС
Subgroup AI (Rosuvastatin, n = 30)	6 (20.0%)	15 (50.0%)	9 (30.0%)
Subgroup AII (Atorvastatin, n = 30)	6 (20.0%)	16 (53.3%)	8 (26.7%)
Subgroup AIII (Simvastatin, n = 30)	8 (26.7%)	12 (40.0%)	10 (33.3%)
MDR1 С3435Т (Group B)			
	3435СС	3435ТТ	3435СТ
Subgroup BI (Rosuvastatin, n = 30)	8 (26.7%)	7 (23.3%)	15 (50.0%)
Subgroup BII (Atorvastatin, *n* = 30)	9 (30.0%)	7 (23.3%)	14 (46.7%)
Subgroup BIII (Simvastatin, n = 30)	7 (23.3%)	8 (26.7%)	15 (50.0%)

At 4-month followed up, in general the rosuvastatin-treated group with *SLCO1B1* C521T polymorphism (AI) exhibited a statistically significant reduction of TC (30.7%, *P* < 0.001) and LDL-C levels (38.0%, P < 0.001). Subgroups with different *SLCOB1* genotypes also showed a significant decrease in TC and LDL-C levels from baseline (P < 0.001). However, in patients with genotype 521CC, there was a smaller decrease in TC (19.6% versus 35.1 and 32.2%, *p* < 0.01) and LDL-C (31.9% versus 41.0% and 37, 9%, *P* < 0.05) than in patients with than those having 521ТТ and 521CT genotypes.

There were significant decreases in levels of TC (26.2%, р < 0.001) and LDL-C (36.9%, р < 0.001) in atorvastatin-treated patients (AII). As with Subgroup AI, patients with different SLCOB1 genotypes all exhibited a significant reduction of TC and LDL-C levels (*p* < 0.01). However, 521CC genotype carriers had much less improvement in TC (19.7% vs 27.3 and 27.1%, *P* < 0.01) and LDL-C (29.5% vs 39.1 and 36.3%, *P* < 0.02) levels than in patients with other genotypes.

**Fig. 2:**
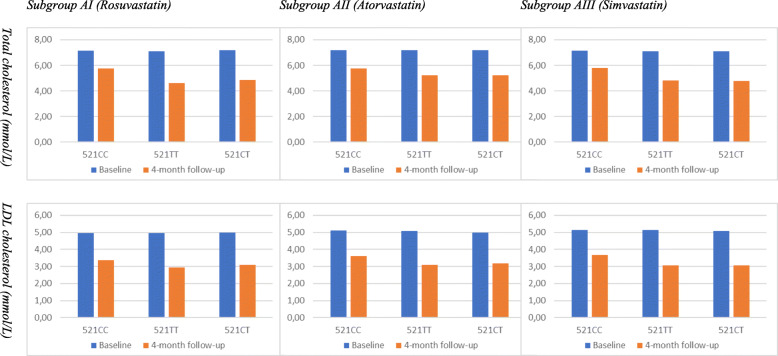
Baseline and follow-up levels of total cholesterol and LDL cholesterol in HLP patients with different SLCO1B1 genotypes who underwent Rosuvastatin, Atorvastatin and Simvastatin therapy (Subgroups AI, AII, AIII)

Patients treated with simvastatin (AIII) showed significantly lower levels of TС and LDL-C than at baseline. While TC levels decreased by 28.9%, LDL-C levels fell by 36.9%, р < 0.001. The genotype-specific trend was similar to those in other two SLCOB1-related subgroups. Although a significant decrease in TC and LDL-C levels (*P* < 0.001) was seen in all patients, regardless of the *SLCOB1* genotype, patients carrying 521CC genotype showed lesser improvement (19.3%) compared with genotypes 521ТТ (32.2%) and 521CT (40.1%). Baseline and follow-up levels of total cholesterol and LDL cholesterol in HLP patients with different SLCO1B1 genotypes who underwent Rosuvastatin, Atorvastatin and Simvastatin therapy (Subgroups AI, AII, and AIII) are shown in Fig. 2 and Table 3.

**Table 3:** Baseline and follow-up levels of total cholesterol and LDL cholesterol in HLP patients with different SLCO1B1 genotypes who underwent Rosuvastatin, Atorvastatin and Simvastatin therapy (Subgroups AI, AII, AIII)

Subgroup	SLCO1В1 genotypes	Total cholesterol (mmol/L)			LDL cholesterol (mmol/L		
		Baseline	4-month follow-up	Change	Baseline	4-month follow-up	Change
Subgroup AI (Rosuvastatin)	521СС (*n* = 6)	7.15 ± 0.14	5.75 ± 0.16*	−19.6%	4.95 ± 0.15	3.37 ± 0.14**	−31.9%
	521ТТ (*n* = 15)	7.10 ± 0.14	4.61 ± 0.15***	−35.1%	4.96 ± 0.17	2.93 ± 0.15***	−41.0%
	521СТ (*n* = 9)	7.17 ± 0.15	4.86 ± 0.17***	−32.2%	4.99 ± 0.18	3.10 ± 0.19***	−37.9%
	Mean (n = 30)	7.13 ± 0.11	4.94 ± 0.14***	−30.7%	4.97 ± 0.17	3.08 ± 0.16***	−38.0%
Subgroup AII (Atorvastatin)	521СС (n = 6)	7.17 ± 0.17	5.74 ± 0.21*	−19.7%	5.12 ± 0.27	3.61 ± 0.28**	−29.5%
	521ТТ (*n* = 16)	7.19 ± 0.14	5.21 ± 0.19***	−27.3%	5.09 ± 0.19	3.10 ± 0.22***	−39.1%
	521СТ (*n* = 8)	7.19 ± 0.15	5.24 ± 0.18***	−27.1%	4.99 ± 0.20	3.18 ± 0.27***	−36.3%
	Mean (n = 30)	7.15 ± 0.18	5.29 ± 0.25***	−26.2%	5.06 ± 0.26	3.19 ± 0.25***	−36.9%
Subgroup AIII (Simvastatin)	521СС (n = 8)	7.16 ± 0.21	5.78 ± 0.22*	−19.3%	5.13 ± 0.19	3.67 ± 0.17**	−28.5%
	521ТТ (*n* = 12)	7.12 ± 0.19	4.83 ± 0.18***	−32.2%	5.14 ± 0.17	3.08 ± 0.18***	−40.1%
	521СТ (*n* = 10)	7.11 ± 0.20	4.79 ± 0.21***	−32.6%	5.09 ± 0.21	3.07 ± 0.16***	−39.7%
	Mean (n = 30)	7.13 ± 0.18	5.07 ± 0.23***	−28.9%	5.12 ± 0.18	3.23 ± 0.23***	−36.9%

Note: *p < 0.05, **p < 0.01, and ***p < 0.001 — significant differences from baseline

For patients with MDR1 С3435Т genotypes (Group B), the following was observed. Subgroup BI (rosuvastatin-treated patients) exhibited a statistically significant reduction of TC (33.3%) and LDL-C levels (38.2%, *P* < 0.001). Although a significant decrease (P < 0.001) was observed in all patients, individuals with 3435CC genotype had slightly higher levels of TC and LDL-C, though statistically insignificant (*P* > 0.05), compared with 3435TT and 3435TC carriers.

There were significant decreases in levels of TC (28.5%, *P* < 0.001) and LDL-C (36.4%, P < 0.001) in Subgroup BII where patients were treated with atorvastatin. Subgroups with different MDR1 genotypes all showed a significant decrease in TC and LDL-C levels from baseline (*P* < 0.001). No statistically significant differences were found between the subgroups (*P* > 0.05).

**Fig. 3:**
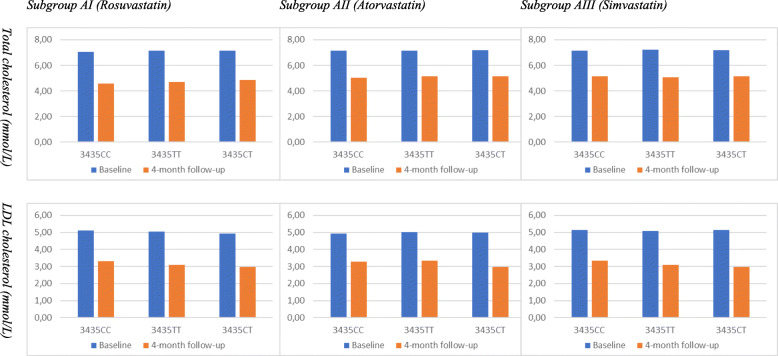
Baseline and follow-up levels of total cholesterol and LDL cholesterol in HLP patients with different MDR1 genotypes who underwent Rosuvastatin, Atorvastatin and Simvastatin therapy (Subgroups BI, BII, BIII)

Patients treated with simvastatin (Subgroup BIII) exhibited a statistically significant reduction of TC (29.6%, *P* < 0.001) and LDL-C levels (38.1%, P < 0.001). In this Subgroup, patients having different MDR1 genotypes showed a significant improvement from baseline (P < 0.001), and no statistically significant differences were found between the subgroups (*P* > 0.05). Baseline and follow-up levels of total cholesterol and LDL cholesterol in HLP patients with different MDR1 genotypes who underwent Rosuvastatin, Atorvastatin and Simvastatin therapy (Subgroups BI, BII, and BIII) are shown in in Fig. 3 and Table 4.

**Table 4:** Baseline and follow-up levels of total cholesterol and LDL cholesterol in HLP patients with different MDR1 genotypes who underwent Rosuvastatin, Atorvastatin and Simvastatin therapy (Subgroups BI, BII, BIII)

Subgroup	MDR1 genotypes	Total cholesterol (mmol/L)			LDL cholesterol (mmol/L		
		Baseline	4-month follow-up	Change	Baseline	4-month follow-up	Change
Subgroup BI (Rosuvastatin)	3435СС (n = 8)	7.08 ± 0.14	4.59 ± 0.17*	−35.1%	5.10 ± 0.17	3.31 ± 0.19*	−35.1%
	3435ТТ (*n* = 7)	7.15 ± 0.17	4.70 ± 0.16*	−34.3%	5.05 ± 0.18	3.11 ± 0.21*	−38.4%
	3435СТ (n = 15)	7.16 ± 0.16	4.88 ± 0.15*	−31.8%	4.92 ± 0.19	2.96 ± 0.20*	−38.8%
	Mean (n = 30)	7.14 ± 0.13	4.76 ± 0.14*	−33.3%	5.00 ± 0.15	3.09 ± 0.17*	−38.2%
Subgroup BII (Atorvastatin)	3435СС (n = 9)	7.13 ± 0.18	5.02 ± 0.22*	−29.6%	4.92 ± 0.19	3.29 ± 0.21*	−33.1%
	3435ТТ (n = 7)	7.14 ± 0.17	5.15 ± 0.24*	−27.9%	5.03 ± 0.18	3.35 ± 0.20*	−33.4%
	3435СТ (*n* = 14)	7.17 ± 0.16	5.14 ± 0.25*	−28.3%	4.98 ± 0.21	2.98 ± 0.22*	−30.1%
	Mean (n = 30)	7.15 ± 0.15	5.11 ± 0.23*	−28.5%	4.97 ± 0.17	3.16 ± 0.19*	−36.4%
Subgroup BIII (Simvastatin)	3435СС (n = 7)	7.16 ± 0.23	5.14 ± 0.31*	−28.9%	5.14 ± 0.20	3.33 ± 0.23*	−35.1%
	3435ТТ (n = 8)	7.21 ± 0.21	5.07 ± 0.32*	−29.7%	5.07 ± 0.19	3.10 ± 0.25*	−38.9%
	3435СТ (n = 15)	7.17 ± 0.17	5.01 ± 0.28*	−30.1%	5.13 ± 0.18	3.12 ± 0.24*	−39.1%
	Mean (n = 30)	7.18 ± 0.19	5.05 ± 0.29*	−29.6%	5.11 ± 0.16	3.16 ± 0.21*	−38.1%

Note: *p < 0.001 — significant differences from baseline

No side effects were reported. The analysis reveals that statin treatment is less effective in lowering TC and LDL-C levels with patients who carry the *SLCO1B1 CC* genotype as compared with patients having TT and CT variants. As for patients with *MDR1* С3435Т genotypes, there were no statistically significant differences between the studied genotypes.

## Discussion

Over a 4-month period, the present study determined the T521C genotype frequency and the dynamics of total cholesterol and LDL cholesterol in patients with hyperlipidemia carrying the *SLCO1B1* C521T and *MDR1* C3435T polymorphisms. The study found that patients carrying the CC genotype of the C521T polymorphism experience a less pronounced decrease in total cholesterol and LDL cholesterol levels during statin therapy than TT and CT genotype carriers, regardless of the drug used. This findings indicates that statin therapy is less effective with T521CC genotype carriers. No statistically significantly were found between patients with TT, CT, and CC genotypes of the C3435T polymorphism.

Genetic polymorphism of OATP1B1 transporters causes changes in the pharmacodynamics of statins [24]. The first retrospective study about the relationship between genetic SLCO1B1 polymorphism and lipid-lowering response to statins was carried out among 66 patients in Japan [26]. The patients received pravastatin, atorvastatin and simvastatin. The results show that individuals with variant C allele had lesser improvement in TC and LDL-C levels compared to TT homozygotes. A study investigating the pharmacodynamics and pharmacokinetics of atorvastatin in 21 Russians found a significant association between SLCO1B1 polymorphism (CC genotype) and a magnitude of LDL reduction [27].

A meta-analysis of 8 studies that encompass 2012 wild-type patients (TT) and 526 carriers of the variant C allele (CT and CC genotypes) found no significant difference in the lipid-lowering efficacy of statins between SLCO1B1 genotypes [28]. However, the study did show an improved lipid-lowering efficacy of simvastatin in TT genotype carriers [28]. Another meta-analysis reveals no association between SLCO1B1 polymorphism and the lipid-lowering efficacy of statins [29]. However, it also shows a stronger effect of the TT variant in the LDL-lowering response to statins and weaker effects in CT and CC genotype carriers. This finding indicates a decrease in the lipid-lowering effect of statins under low OATP1B1 activity. The said meta-analysis also provides evidence of ethnic differences in statins efficacy. For instance, differences seen in the magnitude of LDL reduction between wild (TT) and variant genotype (CT and CC) cases were more pronounced among non-Asian patients.

Current results obtained with the cohort of Russian patients coincide with the latest meta-analysis [20]. In both cases, patients with TT genotype had significantly greater therapy outcomes than CC genotype carriers, regardless of the statins used. At the same time, patients with CT genotype also had substantial improvements in LDL levels, almost as great as with patients carrying the TT genotype.

Given the absence of adverse drug reactions in the present study, no consideration as made for the relationship between SLCO1B1 polymorphism and statin-induced complications. The available literature, however, highlights that variant genotype, especially CC ones, increase the risk of myopathy in patents who take high doses of statins [30, 31]. It is likely that the risk of developing myopathy is more common with lipophilic drugs, such as simvastatin and atorvastatin [32, 33]. These findings urged the Food and Drug Administration (FDA, USA) in 2011 to recommend limiting the use of the maximum doses of simvastatin (80 mg) to keep the risk of myopathy low [34]. Patients with statin-induced myopathy can be classified into three risk groups according to their SLCO1B1 genotype. They are described as normal myopathy risk (TT genotype), intermediate myopathy risk (CT genotype), and high myopathy risk (CC genotype). The Clinical Pharmacogenomics Implementation Consortium Guidelines for SLCO1B1 and Simvastatin-induced Myopathy provide a decision support algorithm for prescribing simvastatin to patients with SLCO1B1*5 genotype [35].

The present study shows no association between statin efficacy and *MDR1* C3435T polymorphism in patients with TT genotype. No statistically significant differences were found in TC and LDL-C reduction between carriers of CT and CC genotypes. The existing research, however, provides contradictory data on the role of C3435T polymorphism in the lipid-lowering response to the statins. An early study of simvastatin-treated Brazilians failed to find such a connection [36]. A study conducted in Russia, on the other hand, revealed that TT genotype carriers had significantly greater reductions in TC and LDL-C levels after simvastatin and atorvastatin than carriers of CT and CC genotypes [37]. No relationship was found between MDR1 polymorphism and rosuvastatin efficacy [37–39]. Another study provides evidence that in the presence of MDR1 polymorphism, statins do not increase the risk of myopathy significantly, but they may cause a substantial increase in the risk of muscle toxicity in C-allele carriers if taken for more than 5 months [25].

Statins are widely used in the treatment of cardiovascular diseases, dyslipidemias and atherosclerosis. At the same time, there is a large variability in the lipid-lowering response to statins, which may be associated with pharmacogenetic characteristics. Studies on polymorphism in OATP and ABC transporters show that these transporters play an active role in the absorption and distribution of statins, the role of ABC transporters is less obvious though.

### Comparisons with other studies and what does the current work add to the existing knowledge

Literature describes a large number of gene polymorphisms that can be considered as potential pharmacogenetic biomarkers of statin-related adverse effects [24]. In practical healthcare, however, pharmacogenetic testing is used to detect just one - the *SLCO1B1* C521T polymorphism, as there is evidence that the carriage of the C521CC allelic variant poses a significant risk of unwanted response from the muscular system [27]. Studies investigating the dependence between the *SLCO1B1* C521T polymorphism and the pharmacological response to statins provide ambiguous results. Some authors report detecting the worst response among patients with the CC genotype [26, 27, 29]. Meantime, other researchers do not indicate such a connection but report an increased lipid-lowering effect of statins in TT genotype carriers [20, 28]. Data on the relationship between *MDR1* C3435T polymorphism and statin therapy are also contradictory [36–38]. The present study confirmed that statins are less effective in patients with the C521CC genotype but failed to demonstrate a statistically significant difference in TC and LDL-C improvement between carriers of different C3435T genotypes.

### Study strength and limitations

The strength of this study is that it investigates the effect of polymorphism in genes encoding the glycoprotein P and the organic anion transporter C on the efficacy and safety of statin therapy, the most commonly used therapy for hyperlipidemia in Russia. The studies were carried out using standard, well-tested methods. The findings expand our understanding of how important the role of gene polymorphism is in promoting the effectiveness of statin therapy. The limitations of this study are associated with a small sample size. Additionally, the present study did not investigate interactions between various gene polymorphisms regarding statin metabolism and efficacy. Nor did it examine the connection between gene polymorphism and statin-induced complications.

## Conclusions

The presence of polymorphisms in genes encoding drug transporters, primarily OATP1B1, can significantly affect the individual response to treatment. Patients with SLCO1B1 521CC genotype are more likely than carriers of 521TT and 521CT genotypes to experience a decrease in the hypolipidemic effect of statins. It is also vital to consider that 521CC genotype carriers have a higher risk of developing myopathy when undergoing a high-dose statin therapy. The MDR1 polymorphism had no substantial impact on the effectiveness of statins. The hypolipidemic efficacy of statins was found to be high in both studied groups. Concerns regarding safety were not established. The most pronounced effects were seen after rosuvastatin. In clinical practice, determining T521C genotypes could be a personalized approach to appropriate statin selection for patients with hyperlipidemia. Meantime, this study found no evidence that determining C3435T genotypes in patients with hyperlipidemia is required. Overall, the results of the present study suggest tailoring statin therapy to the type and dose of medication while taking into account the pharmacogenetic aspects.

## Data Availability

Data will be available on request.
